# Haplotype analysis of *APOE* intragenic SNPs

**DOI:** 10.1186/s12868-018-0413-4

**Published:** 2018-04-19

**Authors:** Vladimir N. Babenko, Dmitry A. Afonnikov, Elena V. Ignatieva, Anton V. Klimov, Fedor E. Gusev, Evgeny I. Rogaev

**Affiliations:** 1 The Federal Research Center Institute of Cytology and Genetics of Siberian Branch of the Russian Academy of Sciences, Center of Neurobiology and Neurogenetics, Lavrentieva str. 10, Novosibirsk, Russia 630090; 2 Novosibirsk State University, Pirogova Str, 2, Novosibirsk, Russia 630090; 3 Vavilov Institute of General Genetics RAS, Gubkina str. 3, Moscow, Russia 119991; 4 Department of Psychiatry, University of Massachusetts Medical School, BNRI, Worcester, MA 15604 USA; 5 Faculty of Biology, Faculty of Bioengineering and Bioinformatics, Lomonosov Moscow State University, Moscow, Russia 119234

**Keywords:** Alzheimer’s disease, *APOE*, ADNI dataset, Haplotype analysis, SNPs, GWAS, PCA, DNA methylation

## Abstract

**Background:**

*APOE* ε4 allele is most common genetic risk factor for Alzheimer’s disease (AD) and cognitive decline. However, it remains poorly understood why only some carriers of *APOE* ε4 develop AD and how ethnic variabilities in *APOE* locus contribute to AD risk. Here, to address the role of *APOE* haplotypes, we reassessed the diversity of APOE locus in major ethnic groups and in Alzheimer’s Disease Neuroimaging Initiative (ADNI) dataset on patients with AD, and subjects with mild cognitive impairment (MCI), and control non-demented individuals.

**Results:**

We performed *APOE* gene haplotype analysis for a short block of five SNPs across the gene using the ADNI whole genome sequencing dataset. The compilation of ADNI data with 1000 Genomes identified the *APOE* ε4 linked haplotypes, which appeared to be distant for the Asian, African and European populations. The common European ε4-bearing haplotype is associated with AD but not with MCI, and the Africans lack this haplotype. Haplotypic inference revealed alleles that may confer protection against AD. By assessing the DNA methylation profile of the *APOE* haplotypes, we found that the AD-associated haplotype features elevated *APOE* CpG content, implying that this locus can also be regulated by genetic-epigenetic interactions.

**Conclusions:**

We showed that SNP frequency profiles within *APOE* locus are highly skewed to population-specific haplotypes, suggesting that the ancestral background within different sites at *APOE* gene may shape the disease phenotype. We propose that our results can be utilized for more specific risk assessment based on population descent of the individuals and on higher specificity of five site haplotypes associated with AD.

**Electronic supplementary material:**

The online version of this article (10.1186/s12868-018-0413-4) contains supplementary material, which is available to authorized users.

## Background

Alzheimer’s disease (AD) is the most frequent case of dementia worldwide, which is manifested by a progressive decline in cognitive function due to loss of neurons, white matter, and synapses. Although it is thought to be caused by progressive accumulation of diffuse and neuritic extracellular amyloid plaques and intracellular neurofibrillary tangles in the brains of AD patients, the etiological mechanisms underlying the neurodegeneration process remain unclear. Since its conception in 2004, Alzheimer’s Disease Neuroimaging Initiative (ADNI, http://www.adni-info.org/) has been searching for associations between MRI brain profiles, biomarkers and clinical symptoms. To date, the significant progress has been made for neuroimaging of the ADNI subjects and in identifying potentially predictive biomarkers for AD [1–6]. Importantly, the whole genome sequencing (WGS) has also been performed for > 800 subjects in ADNI cohort, including AD-patients, individuals with Mild Cognitive Impairment (MCI) and healthy control individuals (CT) (Materials and methods).

Since 1993, the highly significant association of AD with *APOE* ε4 allele has been demonstrated for various ethnic populations [7]. Two missense nucleotide polymorphisms (SNPs) of *APOE*, i.e. rs429358 at codon 112 and rs7412 at codon 158, determine the genotype of *APOE* for ε2, ε3, and ε4 protein isoforms. In particular, *APOE* ε2 represents the major rs429358 variant and minor rs7412 variant (TT haplotype, correspondingly), while *APOE* ε3 is presented by (TC) and *APOE* ε4 by (CC) haplotypes. Notably, it was ascertained that sole rs429358 is the most common AD-associated variant.

*APOE* gene encodes a plasma apolipoprotein protein E that plays a prominent role in lipid metabolism and cholesterol transport in human tissues [8, 9]. Apolipolipoprotein E maintains affinity for receptors involved in the clearance of remnants of very low density lipoproteins [10]. The biological activity of APOE can be altered by modification of its structure. The APOE isoforms, E2, E3 and E4, are metabolically distinct and differ in their affinity for lipoprotein particles and low-density lipoprotein receptors [11, 12]. Possession of the *APOE* ε4 allele, the strong genetic factor for AD, facilitates the Aβ deposition from the presymptomatic stage of AD in a gene-dosage-dependent manner. In contrast, the *APOE* ε2 allele appears to decrease AD risk. [13]. Recently, the *APOE* isoforms are shown to differentially modulate the cellular uptake of Aβ mediated by sortilin related receptor 1 (LR11/SorLA) [14]. Thus, it is also plausible that *APOE* isoforms differentially induce the AD pathology through their cooperation with LR11/SorLA, which is involved in the lysosomal targeting of extracellular amyloid-β (Aβ) [15]. However, the exact molecular mechanism underlying the genetic association of AD with *APOE* [16] remains poorly understood. The presumable DNA methylation shifts for *APOE* alleles in aging may potentially contribute to differential regulation of APOE alleles [17, 18]. Interestingly, the pattern of AD-association with *APOE* varies across human populations. For example, *APOE* ε4 association with AD is lower or even lacking in African-Americans, Hispanic or Yoruban-African populations [19–21]. At the same time, the risk of developing AD in *APOE* ε4 carriers can be modified by other genetic variants, for example, allele G of rs2373115 in gene GAB2 was reported to increase the risk [22].

In this study, we investigated the population dynamics of *APOE* haplotypes and their association with AD development. We also assessed *APOE* methylation profile and found that some intragenic SNPs can be connected to *APOE* DNA methylation shift.

## Datasets and methods

### ADNI data

Data used in the preparation of this article were obtained from the Alzheimer’s Disease Neuroimaging Initiative (ADNI) database (adni.loni.usc.edu). The ADNI was launched in 2003 as a public–private partnership, led by Principal Investigator Michael W. Weiner, MD. The primary goal of ADNI was to test whether serial magnetic resonance imaging (MRI), positron emission tomography (PET), other biological markers, and clinical and neuropsychological assessment can be combined to measure the progression of mild cognitive impairment (MCI) and early Alzheimer’s disease (AD). For up-to-date information, see www.adni-info.org.

Whole genome sequencing data for 809 subjects in VCF format have been downloaded from ADNI web site. It comprised 183 AD-affected individuals, 370 Mild Cognitive Impairment (MCI) ones and 256 of healthy individuals (CT) (http://adni.loni.usc.edu/wp-content/uploads/2010/09/ADNI_WGS_Notice_20130917.pdf). The dataset consists mainly of White individuals (94%). The variant data were called using Illumina’s CASAVA SNP Caller (http://support.illumina.com/sequencing/sequencing_software/casava.html) and are available in VCF format.

### 1000 Genomes data

We prepared a subset of 1000 Genomes data for 3 populations: (a) African (97 individuals from Luhya in Webuye, Kenya (LWK); 88 individuals of Yoruba in Ibadan, Nigeria (YRI); 185 individuals total), (b) East Asian (Han Chinese Southern (CHS0—100 individuals, Han Chinese in Bejing (CSB)—97 individuals, Japanese in Tokyo (JPT)—89 individuals; 286 total); (c) European sample (88 individuals from British in England and Scotland (GBR); 98 individuals from Toscani in Italia (TSI); 94 individuals from Finnish in Finland (FIN); 85 individuals from Utah Residents (CEPH) with Northern and Western European Ancestry; 365 total). The data is available at http://www.internationalgenome.org/data/.

### Association analysis

For association analysis in APOE locus we excluded all genetic variants with allele frequency (MAF) less than 1%, call rates less than 98% or not in Hardy–Weinberg equilibrium (*P* < 10^−4^ in controls). Then we used R-based GenABEL program [23] for assessing the association with AD, using a AD individuals as cases and excluding MCI individuals from analysis. Using the threshold of 5E−8 to select the statistically significant associations with AD phenotype resulted in 27 SNPs from chromosome 19 in *APOE* vicinity (Additional file 1: Table S1; Additional file 2: Fig. S1). Two of these 27 SNPs were located within *APOE* gene (Table 1).

**Table 1:** Two highly significant SNPs within *APOE* gene locus revealed by ADNI GWA analysis

	*P* value genotypes	*P* value alleles
rs769449	8,6E − 15	4,3E − 14
rs429358	4,8E − 18	2,58E − 17

### Haplotype analysis

We used Arlequin ver 3.5 [24], Haploview (https://www.broadinstitute.org/haploview/downloads), PLINK [25] software for assessing linkage disequilibrium and haplotype inferences in *APOE* locus region. In particular, we used ‘haploblocks’ option of Haploview to segregate SNPs into blocks based on ‘tagged’ SNPs [26] algorithm. A block is defined as consequent SNP set over which 95% of informative pairwise comparisons are in strong *LD* [26].

We inferred the most likely gametic phases of 5 locus genotypes using a pseudo-Bayesian approach (ELB algorithm) [24]. Based on the phased haplotype profiles we inferred the ML haplotype phylogenetic tree for 5 SNPs within the *APOE* locus. Due to strong linkage disequilibrium at the region [16], the in silico phasing has proved to be non-ambiguous and thus efficient in this particular case. The pairwise comparison of haplotype frequencies has been carried out by Conventional F-test implemented in Arlequin software [24]. In particular, Average number of pairwise differences between populations P(X,Y) has been calculated, then average pairwise differences within populations has been carried out denoted by P(X). Lastly, the corrected pairwise differences between populations was calculated as P’(X,Y) = (P(X,y) − (P(X) + P(Y))/2) [24]. *P* values have been calculated by Monte-Carlo Method based on 100,000 simulations for each pair.

We used XLStat software for Principal Components analysis (www.xlstat.com).

Haplotype-specific association analysis was performed with Fisher’s exact test against the most common European haplotype (GGATC).

### Phylogenetic analysis

PHYLIP DNAML software was used to build an unrooted phylogenetic tree of observed haplotype sequences.

### Methylation profile analysis

For methylation analysis of *APOE*, we used ENCODE epigenetic profiles for 63 cell lines obtained using Illumina Infinium Human Methylation 450 platform (http://www.genome.ucsc.edu/cgi-bin/hgTrackUi?hgsid=612438305_2wY2cahV1lPNoxcvDm5zHm99hpjz&c=chr19&g=wgEncodeHaibMethyl450) and DNA methylation profiles of human fetal brain [27].

## Results

**Fig. 1:**
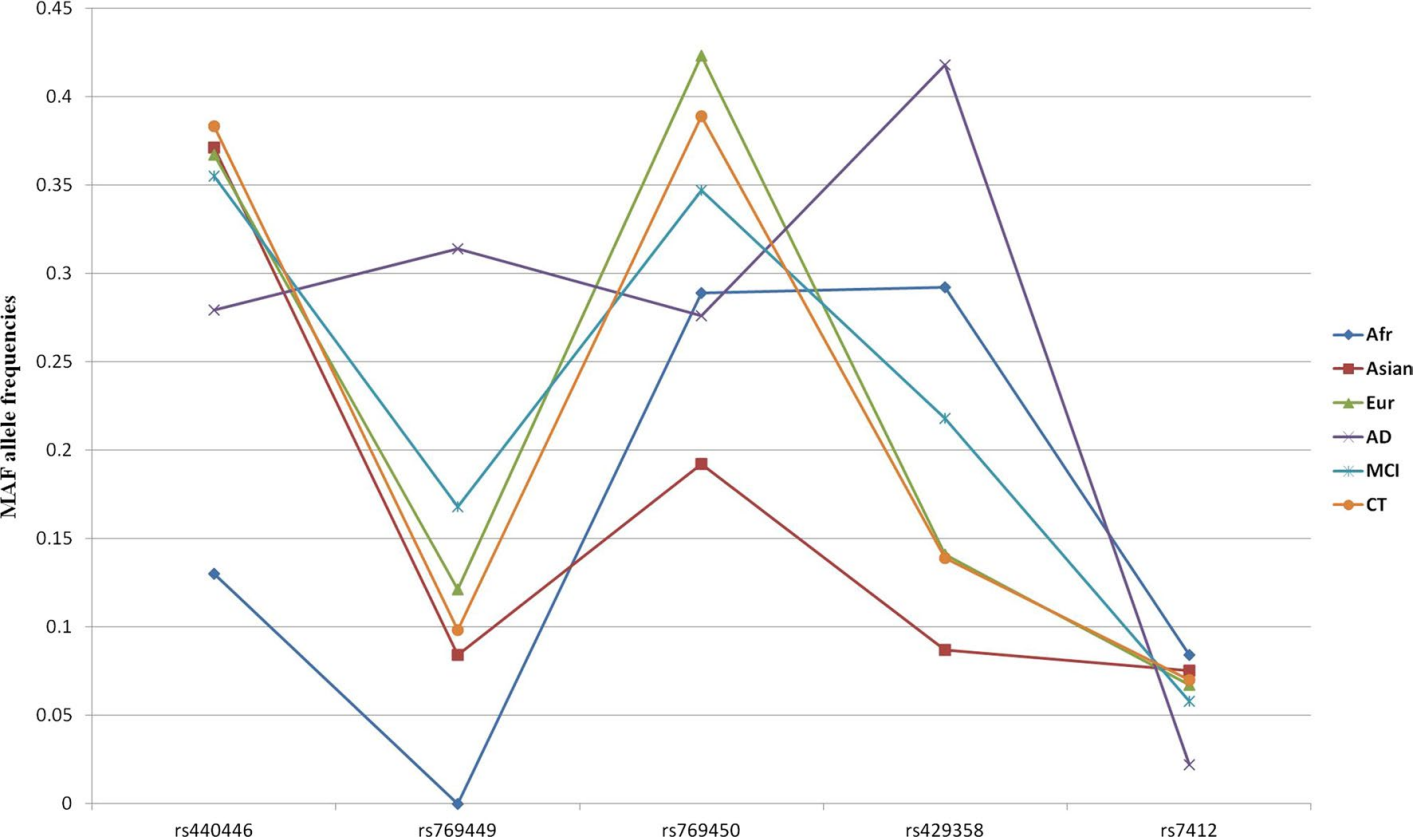
Minor allele frequencies of five SNPs included in analysis. Intronic rs769449 and *APOE* ε4 rs429358 SNPs with significant association to AD are encircled

Analysis of SNP frequencies in *APOE* gene revealed that only five of them are common in human population with frequency > 5% (Fig. 1, Table 2). Three of these SNPs are non-coding variants. The other two (rs429358 and rs7412) are missense variations that define *APOE* ε2, ε3 and ε4 isoforms. We focused on haplotype analysis of these five genetic variants.

**Table 2:** Minor allele frequencies of 5 SNPs included in analysis. SNPs are sorted by chromosomal position. SNPs with significant association to AD in bold

SNP ID	Alleles (MAF 1^st^)	Type	Global MAF	1000G (MAF) samples			ADNI (MAF) samples		
				Afr	Asian	Eur	AD	MCI	CT
rs440446	C/G	Noncoding	0.37	0.130	0.371	0.367	0.279	0.355	0.383
**rs769449**	A/G	Noncoding	0.06	0.000	0.084	0.121	*0.314*	0.168	0.098
rs769450	A/G	Noncoding	0.33	0.289	0.192	0.423	0.276	0.347	0.389
**rs429358**	C/T	Missense	0.15	0.292	0.087	0.141	*0.418*	0.218	0.139
rs7412	T/C	Missense	0.07	0.084	0.075	0.067	0.022	0.058	0.070
Sample size				185	286	365	183	370	256

In line with previous reports, we found multiple SNPs in *APOE* locus to have a significant association to AD (Additional file 1: Table S1; Additional file 2: Fig S1). However, only two of the above five SNPs in *APOE* gene (rs769449 and rs429358) show statistically significant association with AD (Table 1).

**Fig. 2:**
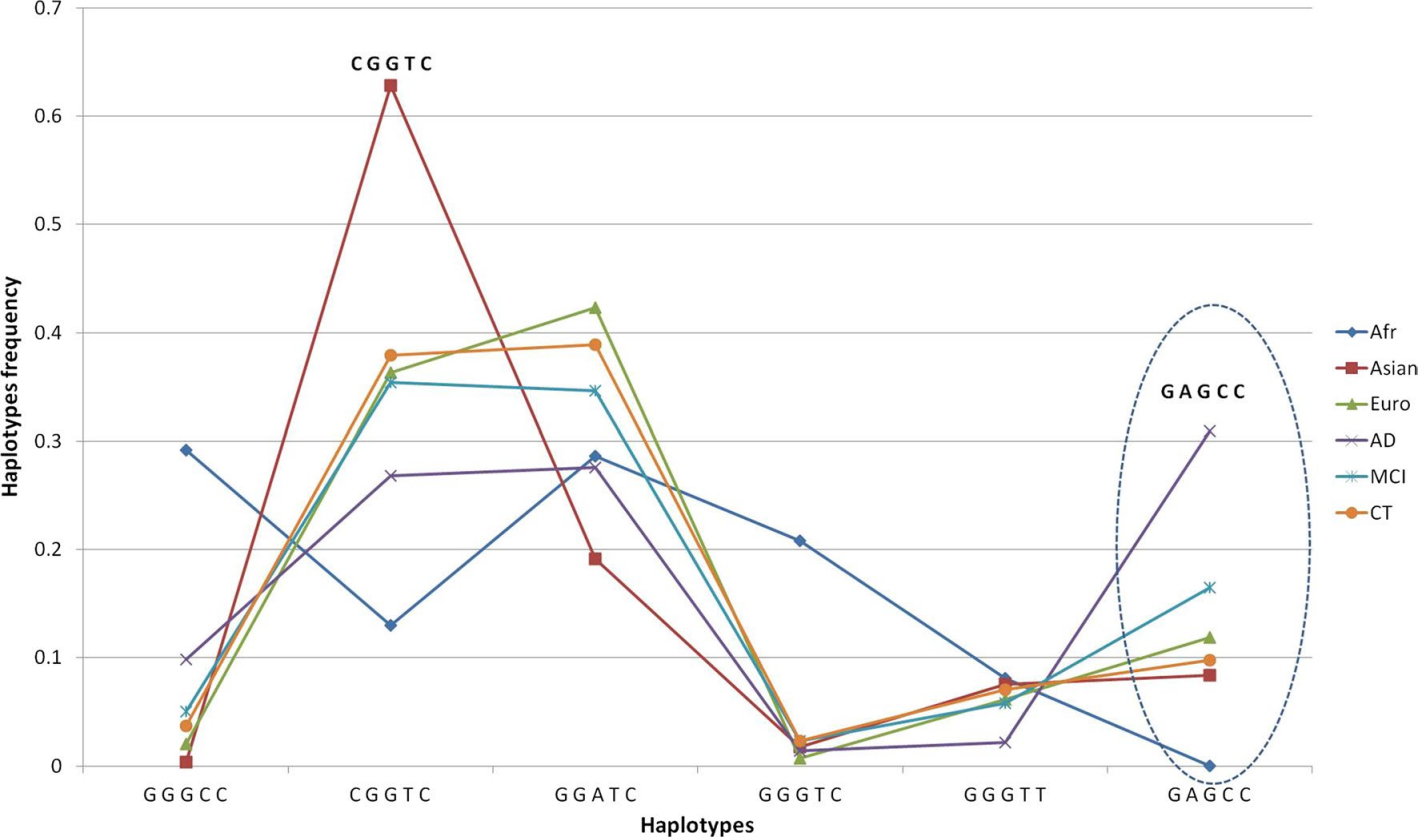
Haplotype frequencies in human populations and ADNI cohort. X-axis labels represent allelic status of 5 SNPs (rs440446, rs769449, rs769450, rs429358,rs7412; Table 2) in *APOE locus*. Haplotype associated with *APOE* ε4 are in bold italic

Next, we conducted a haplotype-based analysis of the five SNPs. Pairwise linkage analysis revealed that rs769449 and rs429358 are linked in Asian individuals (r2 = 0.956), but have a lower linkage in European sample (r2 = 0.828), while rs769449 is not polymorphic in Africans. Further, using Arlequin software, we assessed haplotype frequencies (haplotypes with f > 0.01) for six human cohorts (Fig. 2, Table 3).

**Table 3:** Haplotype frequencies in six human cohorts (haplotypes with *f *> 0.001). AD/MCI vs CT:GGATC (last column) represents the association of the haplotype with AD when compared to most common European haplotype (Fisher’s exact test); haplotypes with zero frequencies are excluded from analysis

Haplotype	# added CG (positions)	*APOE* Allele	1000G			ADNI				
			Afr	Asian	Eur	AD	MCI	CT	AD vs CT:GGATC *P* value	MCI vs CT:GGATC *P* value
G G G CC	3 (1,4,5)	ε4	0.292	0.0035	0.0205	0.0984	0.05	0.0371	1.6e-5	0.1975
C G G T C	1 (5)	ε3	0.13	0.628	0.363	0.268	0.354	0.379	1	0.7382
G G A T C	2 (1,5)	ε3	0.286	0.191	0.423	0.276	0.346	0.389	NA	
G G G T C	2 (1,5)	ε3	0.208	0.0175	0.00685	0.0137	0.023	0.0234	0.7979	0.8491
G G G T T	1(1)	ε2	0.0811	0.0752	0.0616	0.0219	0.0581	0.0703	0.039	0.8065
G A G C C	3 (1,4,5)	ε4	0	0.0839	0.119	0.309	0.165	0.0977	2e-13	0.001
G G A T T	1 (1)	ε2	0.0027	0	0	0	0	0		
C G A T C	1 (5)	ε3	0	0.00175	0	0	0	0		
C G G T T	0	ε2	0	0	0.00411	0	0	0		
G A G C T	2 (1,4)	ε3	0	0	0.00137	0	0	0		
G A G T C	2 (1,5)	ε3	0	0	0	0.00273	0.00135	0		
C G G C C	2 (4,5)	ε4	0	0	0	0.0082	0	0.00391	0.3419	
C A G C C	2 (4,5)	ε4	0	0	0	0.00273	0.00135	0		
G G A C C	3 (1,4,5)	ε4	0	0	0	0	0.00135	0		
Total haplotypes			370	572	730	366	740	512		
Sample size			185	286	365	183	370	256		

**Fig. 3:**
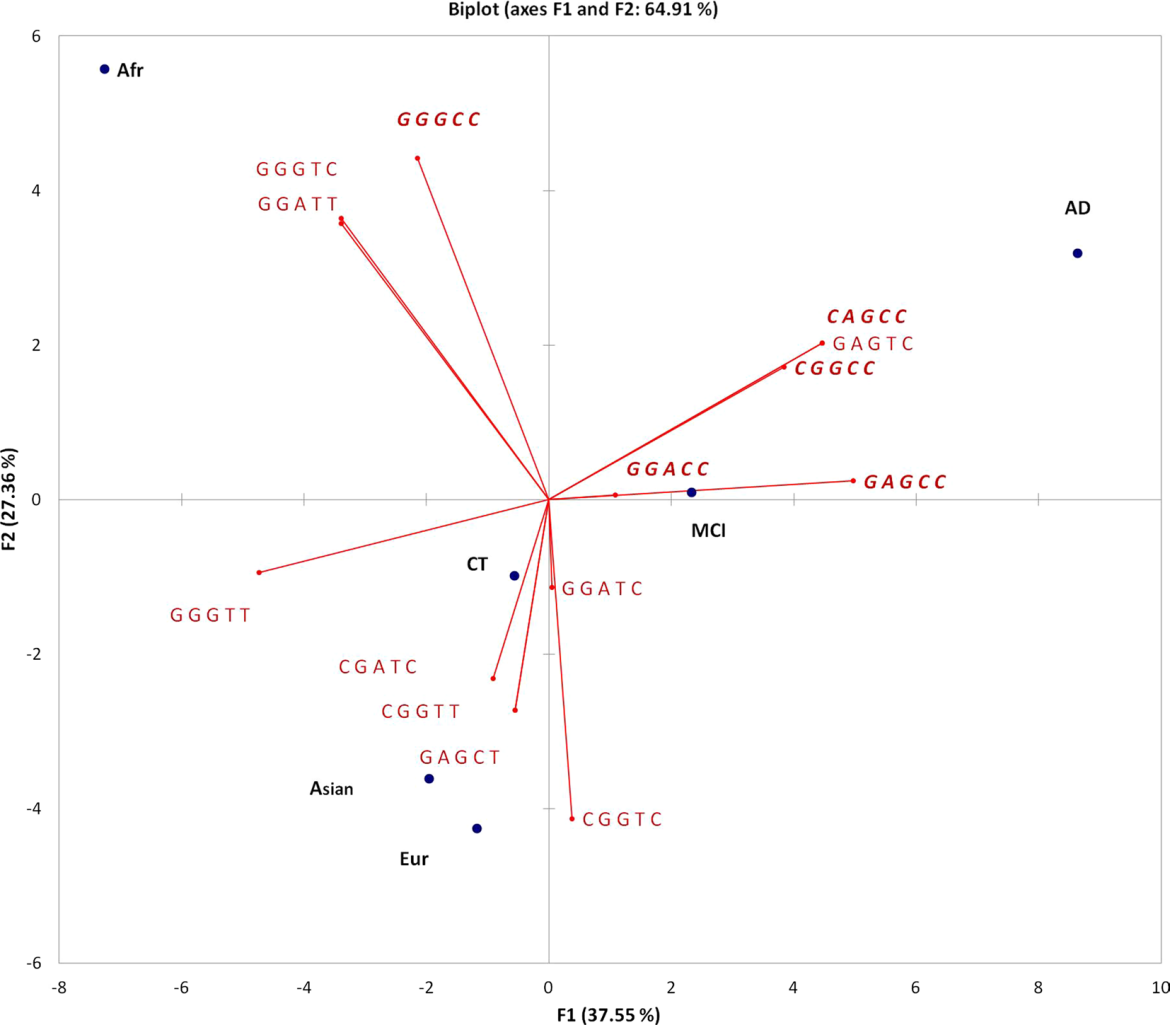
Principal Components analysis based on the haplotype frequencies distribution across 6 populations (Afr, Ori, Eur, AD, MCI, CT). Haplotype encoding corresponds to Table 2. AD—associated haplotypes are marked by the bold italic type

Analysis of these data demonstrated that haplotype frequency profiles are distinct in human populations (Fig. 3, Table 3). This is supported by statistical analysis, which demonstrated that each pair of populations/cohorts are significantly different (*P* value < 1e−4), except, for 1000 Genomes European population versus ADNI Control cohort (Table 4). The most common haplotypes (which are presented by *APOE* ε3-bearing alleles) have different frequencies across populations: GGGTC is almost absent in Europeans (< 1%) and Asians (< 2%), but common in Africans (20%), CGGTC is present in 62% of Asians, but at lower frequencies in Europeans (36%) and Africans (13%). We also observed that two most common ε4—bearing haplotypes have a clear population-specific patterns. GAGCC is present exclusively in Asian and European populations and absent in African population. In contrast GGGCC is the only ε4—bearing haplotype presented in 29% of African individuals, but occurs at low frequencies in Asian and European groups (< 2%). Surprisingly, a protective *APOE* ε2 allele is presented almost exclusively by a single haplotype GGGTT in all human populations with 6–8% frequency. Thus, this allele has a lower population diversity.

**Table 4:** *P* value for pairwise comparison of populations based on their haplotype frequencies [24]. ADNI Control group and European population don't significantly differ

	Afr	Ori	Eur	AD	MCI
Ori	< 10E − 4				
Eur	< 10E − 4	< 10E − 4			
AD	< 10E − 4	< 10E − 4	< 10E − 4		
MCI	< 10E − 4	< 10E − 4	< 10E − 4	< 10E − 4	
CT	< 10E − 4	< 10E − 4	*0.5*	< 10E − 4	0.00909

**Fig. 4:**
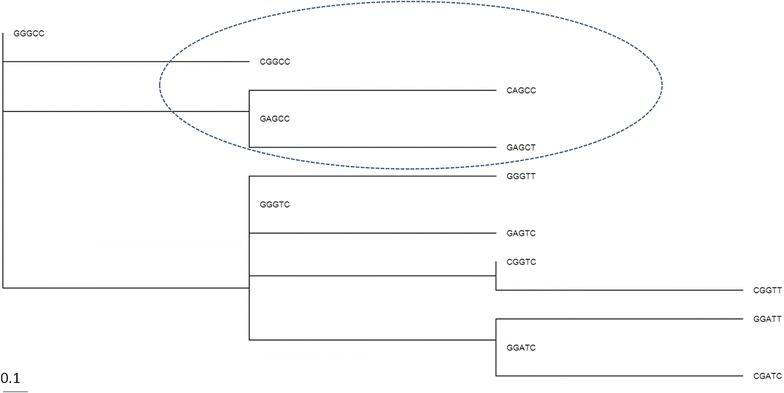
Phylogenetic tree of *APOE* haplotypes. GAGCC is the disease haplotype specific to Europeans (Table 3). The AD-associated haplotype subset is encircled

Phylogenetic analysis of *APOE* haplotypes revealed that *APOE* ε4 haplotype GGGCC, which is African-specific, is most likely the ancestral variant (Fig. 4). This suggests that a common *APOE* ε3 allele was distributed in human populations after the split with other archaic hominins.

Comparing nucleotide content in the two ε4-bearing haplotypes (GGGCC and GAGCC) we observed that allele G of second SNP (rs769449) separates Africans from individuals of European and Asian ancestry. When we compared these two haplotypes to the most common European haplotype (GGATC), we found that both are significantly associated with AD (Fisher’s exact test *P* value < 1e−12 and *P* value < 1e−4), but only GAGCC is associated with MCI. Altogether the data suggests, the state of this SNP might have a modifying effect on ε4-associated AD/MCI risk development with African-specific allele G being potentially protective, in particular, in African populations.

**Fig. 5:**
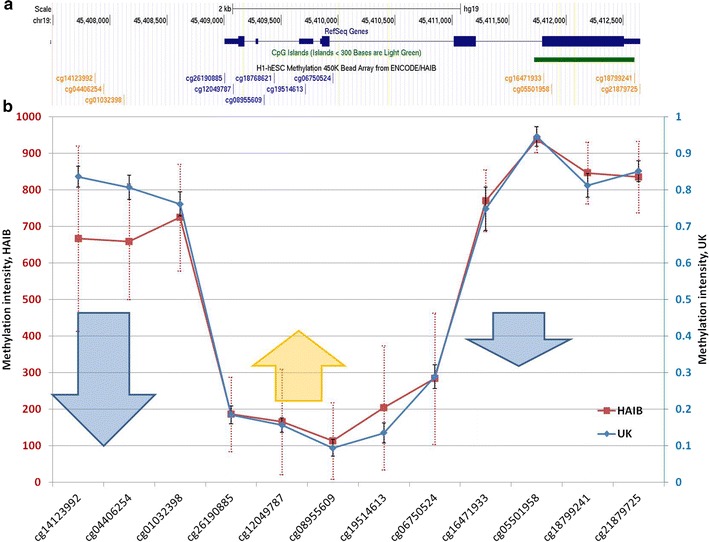
Methylation profile of the *APOE* locus. **a** Genomic location of Illumina Methyl 450 bead array probes; **b** methylation profile of *APOE* gene based on two methylome projects. 63 HAIB cell lines (HAIB ENCODE methylation data), and 179 fetal brain samples [27] were used. Vertical dotted bars correspond to standard deviation of methylation score. Arrows indicate age related methylation drive [18]

This SNP is non-coding and therefore might have a regulatory effect on *APOE*. Potentially, A vs G allele in rs769449 can modify the epigenetic state in the *APOE* gene region. Supporting this hypothesis, we observed a robust H3K4Me3 signal using ChIP-seq data in this rs769449-containing region (Fig. 5; encircled) that is common mark of open chromatin. We assessed methylation profile of *APOE* locus based on ENCODE HAIB methylation data performed using Illumina Human Methylation 450 K Bead Arrays (Fig. 5) [18, 27]. While the methylation profile is U-shaped, the region from TSS down to exon 4 is highly sensitive to methylation [18], and comprises a range of transcription factor binding sites (Additional file 1: Table S2). The methylation rate of this region, which includes the SNP rs769449, is anticorrelated with *APOE* expression rate and is significantly associated with aging [18]. It is also located 78 bp downsteam to second *APOE* exon. The methylation state in this region is changed in aging and associated with *APOE* dysfunction [18]. The rs769449 context is (gGc) and, when turning to A, one of the methylation sites drops out, thus possibly altering intragenic methylation profile. A set of transcription factor binding sites in the areas of SNPs rs769449 also implies its possible regulatory effect (Additional file 1: Table S2).

**Fig. 6:**
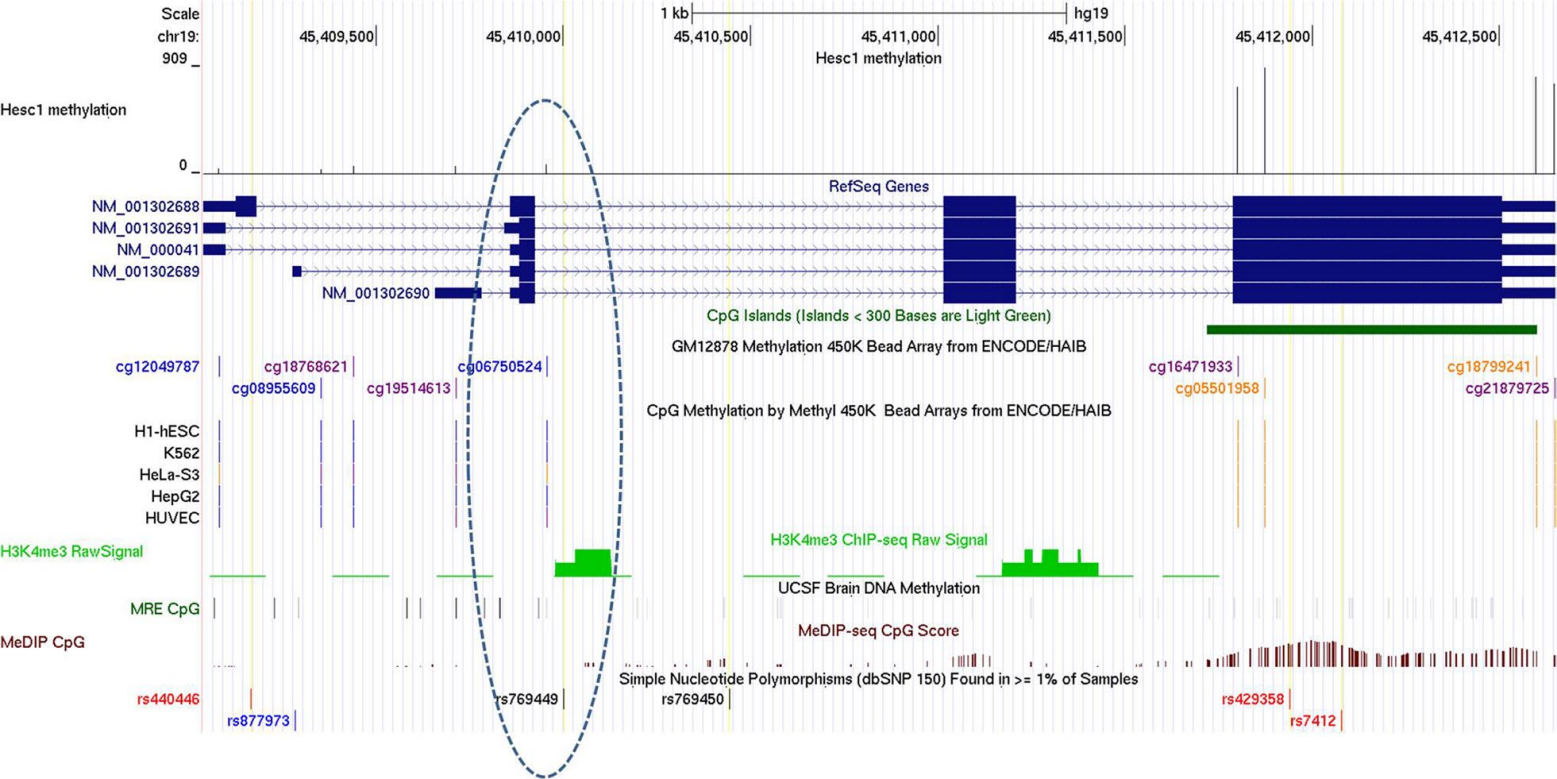
UCSC genome browser screenshot depicting active H3K4me3 spot in the vicinity of rs769449 (encircled) along with previously associated with *APOE* ε4 cg06750524 methylation status [18]

It is worth noting that at least three out of five SNPs affect the CG dinucleotide content in *APOE* gene. *APOE* ε4 bears two CG dinucleotides mediated by rs429358 (minor allele) and rs7412 (major allele) that reside in the CpG island of exon 4 (Fig. 5) [18, 28]. rs769450 does not affect CG content (Table 2), while rs440446, the first target haplotype SNP meditates the CG dinucleotide arisen by minor allele, similar to the last ones,. Thus, the *APOE* ε4-bearing haplotypes maintain the largest number of CG dinucleotides within *APOE* (Table 3). Notably, rs769449 mediates CG dinucleotide in the inverse strand. It is resided within hotspot of H3K3me3 region (Fig. 6; encircled), and its C→A transition might affect the binding site of the transcription factor (Additional file 1: Table S1). Notably, the target Illumina Methylation 450 array CpG site cg06750524 located close to rs769449 (Fig. 6; encircled) methylation status is highly associated with *APOE* ε4 allele: it was reported that its methylation rate is higher for the minor “disease” allele [18].

## Discussion

*APOE* gene maintains the highest genetic association with AD reported to date. However, the association is ethnic- dependent, e.g., the evidence for AD-association with *APOE* is lower for African-Americans, Hispanic or Yoruban-African populatuion [19–21]. We have demonstrated that frequencies of *APOE* haplotypes a significantly different in human populations (Fig. 2, Table 4). Specifically, the context of *APOE* ε4, which is the AD risk allele, drastically differs in populations (Fig. 3, Table 3). In particular, the two haplotypes for AD-associated *APOE* ε4 variant are GGGCC for African, and CAGCC for European and Asian individuals.

Sequence analysis of the chimpanzee *APOE* gene showed that it is most closely related to human ε4-type haplotypes, differing from the human consensus sequence at 67 synonymous (54 substitutions and 13 indels) and 9 nonsynonymous fixed positions [29]. Our analysis showed further that haplotypes defining the ε3 and ε2 alleles are derived from the ancestral ε4 s and that the ε3 group of haplotypes have increased in European and Asian populations.

The issue of ancestry of *APOE* ε4 allelotype has been widely discussed [30], and it has been established that the C→ T variant for ε3 allele arose after primate radiation [30]. Its relatively rapid expansion could be attributed to converging to meat diet in ancient human populations [30]. The data suggest also that specific *APOE* haplotypes might have protective effect against AD development potentially via epigenetic reprogramming of *APOE* due to CpG emergence/dropout [18, 28]. Altogether, our data demonstrated that ethnic genetic background defines significant differences in haplotypes for AD- risk alleles in human populations that may potentially be additional factor modifying risk for AD.

## Additional files

**Additional file 1: Table S1.** 27 significant SNPs in the APOE region. **Table S2**. Transcription factor binding sites annotation related to SNPs analysed.

**Additional file 2: Fig. S1.** Output of GenABEL [23] program underlining chromosome 19 APOE region significance on ADNI sample.

## Abbreviations

AD: Alzheimer’s disease
LOD: Logarithm of odds
SNPs: Single nucleotide polymorphisms
