# Supplementary material for: Haplotype analysis of APOE intragenic SNPs
Source: BMC Neurosci. 2018 Apr 19;19(Suppl 1):16. doi: 10.1186/s12868-018-0413-4 (PMC5998902; doi:10.1186/s12868-018-0413-4)
Supplement: Supplementary file 2 — Additional file 2: Fig. S1. Output of GenABEL [23] program underlining chromosome 19 APOE region significance on ADNI sample. [file 12868_2018_413_MOESM2_ESM.doc]

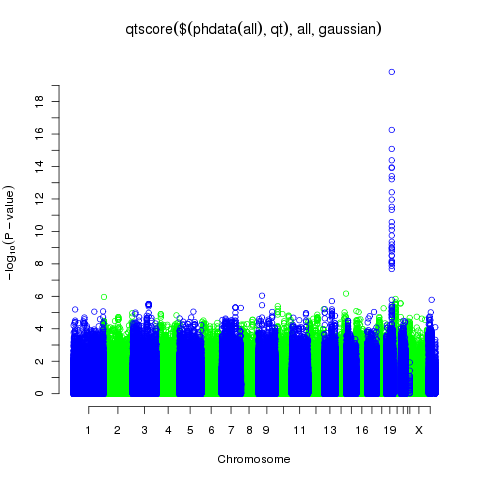


Figure S1. Significant association of APOE locus on chromosome 19 with AD in ADNI sample demonstrated by GenABEL program [1].

1. Aulchenko YS, Ripke S, Isaacs A, van Duijn CM. GenABEL: an R library for

genome-wide association analysis. Bioinformatics. 2007 May 15;23(10):1294-6
